# Empowering Hand Rehabilitation with AI-Powered Gesture Recognition: A Study of an sEMG-Based System

**DOI:** 10.3390/bioengineering10050557

**Published:** 2023-05-06

**Authors:** Kai Guo, Mostafa Orban, Jingxin Lu, Maged S. Al-Quraishi, Hongbo Yang, Mahmoud Elsamanty

**Affiliations:** 1 School of Biomedical Engineering (Suzhou), Division of Life Sciences and Medicine, University of Science and Technology of China, Hefei 230026, China; 2 Suzhou Institute of Biomedical Engineering and Technology, Chinese Academy of Sciences, Suzhou 215163, China; 3 Mechanical Department, Faculty of Engineering at Shoubra, Benha University, Cairo 11672, Egypt; 4 School of Mechanical and Electrical Engineering, Changchun University of Science and Technology, Changchun 130001, China; 5 Faculty of Engineering, Thamar University, Dhamar 87246, Yemen; 6 Mechatronics and Robotics Department, School of Innovative Design Engineering, Egypt-Japan University of Science and Technology, Alexandria 21934, Egypt

**Keywords:** flexible robot, hand rehabilitation, rope-driven, sensory stimulation, wearable device

## Abstract

Stroke is one of the most prevalent health issues that people face today, causing long-term complications such as paresis, hemiparesis, and aphasia. These conditions significantly impact a patient’s physical abilities and cause financial and social hardships. In order to address these challenges, this paper presents a groundbreaking solution—a wearable rehabilitation glove. This motorized glove is designed to provide comfortable and effective rehabilitation for patients with paresis. Its unique soft materials and compact size make it easy to use in clinical settings and at home. The glove can train each finger individually and all fingers together, using assistive force generated by advanced linear integrated actuators controlled by sEMG signals. The glove is also durable and long-lasting, with 4–5 h of battery life. The wearable motorized glove is worn on the affected hand to provide assistive force during rehabilitation training. The key to this glove’s effectiveness is its ability to perform the classified hand gestures acquired from the non-affected hand by integrating four sEMG sensors and a deep learning algorithm (the 1D-CNN algorithm and the InceptionTime algorithm). The InceptionTime algorithm classified ten hand gestures’ sEMG signals with an accuracy of 91.60% and 90.09% in the training and verification sets, respectively. The overall accuracy was 90.89%. It showed potential as a tool for developing effective hand gesture recognition systems. The classified hand gestures can be used as a control command for the motorized wearable glove placed on the affected hand, allowing it to mimic the movements of the non-affected hand. This innovative technology performs rehabilitation exercises based on the theory of mirror therapy and task-oriented therapy. Overall, this wearable rehabilitation glove represents a significant step forward in stroke rehabilitation, offering a practical and effective solution to help patients recover from stroke’s physical, financial, and social impact.

## 1. Introduction

Stroke has become a major contributor to human death globally, with over 10 million people suffering from it annually. This debilitating disease affects the lives of patients and their families, significantly reducing the quality of life of all involved [1]. Despite progress in stroke treatment and increased survival rates in recent years, there is still a critical need for advanced rehabilitation methods to speed up recovery and improve motor function in post-stroke patients. This is where the concept of assistive devices in rehabilitation comes into play. Stroke can cause severe damage to the central nervous system, resulting in various physical impairments and limitations. One of the most common injuries in stroke patients is the loss of wrist motion, a crucial joint connecting the hand and the arm. As such, rehabilitation training for patients with stroke and wrist injuries is essential for improving their overall health and well-being [2,3]. With the help of specialized rehabilitation techniques and assistive devices, stroke patients can regain some of their motor abilities and improve their quality of life.

The traditional approach to rehabilitation, which relies on one-on-one training involving a patient and a therapist, has proven ineffective and cost-prohibitive in today’s world. With the outbreak of the COVID-19 epidemic, face-to-face therapy sessions in hospitals had to be suspended, making the traditional approach to rehabilitation untenable [4]. To address this issue, rehabilitation robots have emerged as a solution by offering home-based rehabilitation programs. These programs allow patients to easily access rehabilitation training from the comfort of their own homes, reducing the need for face-to-face sessions during the pandemic [5]. Using assistive robots in rehabilitation has numerous advantages, including improved safety, efficiency, flexibility, and practicality. Many countries have recognized the need to use technology to mitigate the impact of disabilities and improve the quality of life of people with disabilities [6]. As a result, more people with disabilities rely on assistive devices to carry out daily activities and participate more actively and productively in community life [7]. However, assistive devices and technologies are still limited in many low- and middle-income countries. Only 5–15% of people who require these devices have access to them due to low production, limited quality, a lack of trained personnel, and prohibitive costs [8]. The need for affordable and accessible assistive technologies remains a critical challenge in many parts of the world, and more needs to be done to address this disparity.

The availability of assistive devices is critical for many countries’ economic and social development. It enables people with disabilities to participate in community activities, work, and earn an income, reducing the poverty levels [9]. As a result, rehabilitation and the development of assistive devices for stroke patients have become urgent and critical issues for many countries. To design effective rehabilitation assistive devices, it is crucial to understand their ideal properties for hand function and simulate simple and complex hand movements. The device should be comfortable, affordable, portable, and easy to use in clinical rehabilitation settings and at home [10]. A timely and efficient rehabilitation is critical, as prolonged gaps between therapy sessions can negatively impact rehabilitation. Assistive devices have been shown to speed up the rehabilitation process, and incorporating complementary therapies, such as mirror therapy and task-oriented therapy, can further enhance the effectiveness of rehabilitation [11,12]. Overall, the development and availability of assistive devices for rehabilitation are essential for promoting economic and social development, reducing the poverty levels, and improving the quality of life of people with disabilities.

In 2018, Randazzo et al. proposed a novel hand exoskeleton design to assist and rehabilitate individuals with impaired movement function and improve their quality of life. The device is wearable, portable, and does not impede hand mobility. It utilizes an EEG brain–machine interface to control hand movements through pattern detection. Finger actuation is achieved using Bowden cables, a linear actuator, with each finger having an active DOF for flexion and extension. The user can passively control abduction and adduction. The device is cost-effective, utilizing off-the-shelf parts as well as 3D printing and laser cutting. The actuator is housed in a closed box mounted on the chest, connected to the fingers via Bowden cables. The device is powered by a lithium–polymer (LiPo) battery, capable of continuous operation for up to 3 h [13].

In 2020, Butzer T. et al. designed the RELab tenoexo, a fully wearable, soft hand exoskeleton for daily activities, It consists of a hand module attached to the hand and a backpack containing electronic components, motors, and batteries. The backpack and hand module are connected by a force transmission system based on Bowden cables and can be attached by a clip mechanism. In the hand module, three DOF achieve dynamic, precision, and lateral grasping: a combination of 2–5-digit actuators for flexion/extension, an individual actuator for the thumb flexion/extension, and a manual lateral and oppositional pad for the thumb. Two DC motors are fed forward and controlled to drive the flexion/extension of the fingers and thumb through gear-rack mechanisms. The Myo armband (a wireless sEMG sensor for monitoring) is used [14].

In 2022, Marek Sierotowicz and colleagues from the Robotics and Mechatronics Department at the German Aerospace Center (DLR) designed an EMG-driven machine learning-controlled soft glove for assisting grasp and rehabilitation. The glove can assist the flexion and extension of the index finger and thumb through a tendon-driven system. The two actions, thumb and finger flexion, are independently assisted by electric motors that pull and release their respective tendon lines. The glove has a control system that recognizes attention through sEMG [15].

Despite these efforts, we think that the shape of the device and the fact that it has to be carried on the chest or a backpack still have some negative implications for the user, especially for stroke patients who usually suffer from a lack of self-confidence within a community, which can affect the progress of the training beside the short time of use of 3 h. In addition, the size and the weight of the device have a negative effect on the comfort of the user.

This article presents a hand rehabilitation system with a soft glove that supports mirror, task-oriented therapies, and neural plasticity. The actuated glove, integrated with a linear actuator, provides the driving force for the affected hand during rehabilitation training. The sEMG sensor enables cooperation between both hands and was used to control the actuated glove to let the affected and the non-affected hands move simultaneously. An integrated sensor measures the bending angle and the progress of the rehabilitation programmer. A machine learning algorithm was developed to classify the sEMG gestures as a control command for the actuated glove. This gives the affected hand the suitable driving force to perform the corresponding motions. Our glove is superior to previous devices because of its lightweight, the soft material used for its fabrication, its long-lifetime batteries that can support 4–5 h of use, and its low cost, which is a good advantage for low-income countries, as discussed before. The device is built so that all of its parts are hidden under the shoulder and under the clothes, and the only visible part is the glove placed on the wrist, which positively affects the user’s self-confidence.

Moreover, our glove can train each finger alone to perform flexion and extension with the integrated sensor to measure the angle of bending that reflects the progress of the rehabilitation program. Finally, our glove is based on two verified therapy theories, mirror therapy and task-oriented therapy, using sEMG. The telecooperation between the affected and the unaffected hand has also a good effect on the progress of the therapy training.

## 2. Materials and Methods

### 2.1. Related Work

#### 2.1.1. Hand Design and Actuation

The research and development of hand rehabilitation equipment are still in their infancy. Most exoskeleton-hand robots focus on improving finger joints and increasing muscle strength, ignoring the vital role of active brain participation and the sensory function input into the motor function. Therefore, the effect of fine motor rehabilitation on hand function is not considered. At present, the hand-function rehabilitation robots design adopts an exoskeleton structure, which mainly provides a driving force through motors and pneumatics to complete flexion and extension of the fingers and joints. Four main driving methods have been used for hand-function rehabilitation robots. They mainly include a motor drive, a pneumatic artificial muscle drive, a memory alloy drive, and a lasso drive. Because the lasso drive has good flexibility and linearity, we used a lasso drive to design hand-functional rehabilitation robots.

#### 2.1.2. Recognition Methods of the sEMG Signals

The electrical voltages in sEMG signals range from −5 to +5 (mV) and are influenced by both the movements performed and the muscle extraction and contraction level. The continuous availability and variability of the signal can be measured using a suitable detection element [16,17,18,19,20,21]. These signals have significant potential in rehabilitation, where they can be combined with an appropriate recognition system. However, given that the sEMG signals traverse multiple tissues and muscles before being acquired from human muscles [22], they are susceptible to interference from crosstalk, obstructions, and noise. Different machine learning and A.I. techniques have been used to classify and recognize the EMG signals. The classifier can be a Supported Vector Machine (SVM) [23], the k-nearest neighbor (kNN) algorithm [24], linear discriminant analysis (LDA) [25], and a neural network (N.N.) [26], with different classification accuracy (C.A.) and complexity for different methods.

Using a neural network with a backpropagation or fuzzy recognition system [27,28] may not be optimal due to its slow learning in many cases. In the EMG-decoding window length study, a mean testing performance of 94.21% ± 4.84% after voting was demonstrated through visual inspection [29]. In another study, supervised feature extraction techniques achieved initial accuracy rates of 91.54% and 80.40%, with additional accuracy rates of 91.71% and 91.44% [30].

A highly accurate decoding and classification method for sEMG signals was developed in this research, which can be used as mirror therapy to mirror the motion from the non-affected hand in the affected hand. The wearable glove developed in this study can perform 16 desired motions, including 6 task-oriented therapy-based motions, and is cost-effective and comfortable. This glove can be used for home-based rehabilitation and is not limited to clinical environments. The individual finger movement and the cooperation of multiple fingers can be trained using this glove. The system design and fabrication process, as well as the methodology used in the research, are described in this article, which begins with sEMG implementation and experiment details in the mirror therapy scenario and ends with data preprocessing and classification.

### 2.2. Design and Manufacture of the Hand Robot

**Figure 1:**
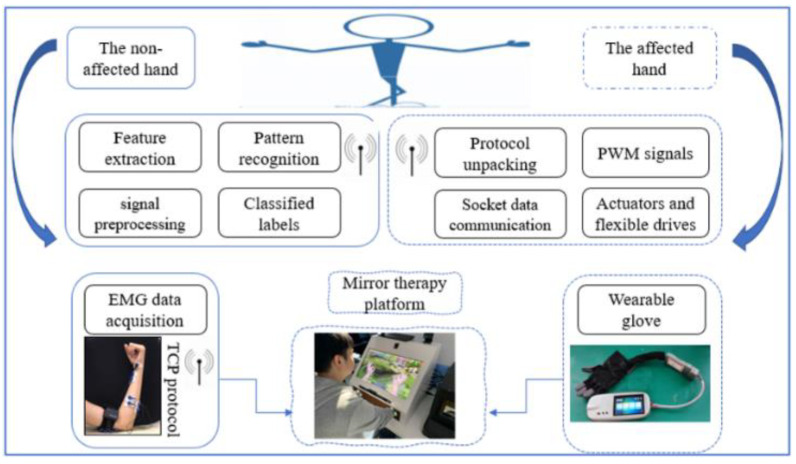
Flexible Hand Rehabilitation Robot with a Linear Actuator and an sEMG Sensor.

This study aimed to introduce a wearable rehabilitation glove based on flexible lasso transmission driven by a flexible rope and a linear actuator. The glove was designed to assist patients with hand impairments in the rehabilitation process and enable them to perform routine activities in daily life, which can boost their confidence and promote their independence. As shown in Figure 1, the glove can simulate the functions of the human hand and facilitate the recovery of patients with various hand injuries or conditions.

The flexible-rope driving method used in the glove design provides several advantages, including a simple structure, a lightweight design, substantial flexibility, comfortable wearing, and convenient use. The flexible rope acts as a transmission mechanism that can transmit power from the linear actuator to the glove without a significant loss of power. This design enables the glove to provide the necessary driving force to assist the patient’s hand movements during the rehabilitation process, while also ensuring the patient’s comfort and safety.

#### 2.2.1. Host Machine for the Hand Rehabilitation Robot

The lightweight design of the glove allows patients to wear it for extended periods without feeling uncomfortable or fatigued, making it suitable for long-term use. Additionally, the flexible lasso transmission provides a substantial range of motion, allowing patients to perform various hand movements without feeling restricted or inhibited. This range of motion is essential in promoting hand function and mobility recovery, which can be challenging for patients with hand injuries or conditions.

**Figure 2:**
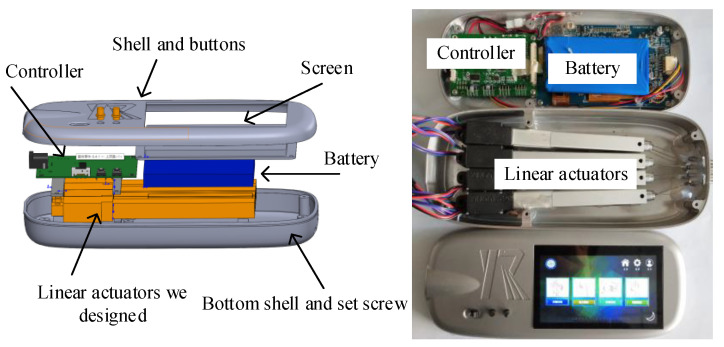

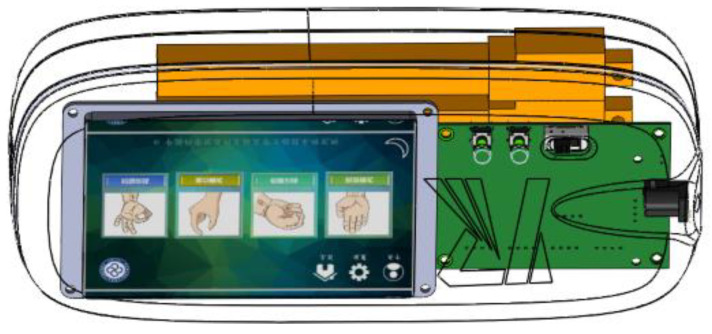
Main module of the hand rehabilitation robot.

The wearable glove presented in this study is primarily composed of a main control module, a separation module, a glove connection module, and wearing gloves, as illustrated in Figure 2. The portable, wearable robot main control module comprises five linear actuators, a battery, hardware, and a screen, all housed within a compact structure. The separator module effectively combines the central control module and the rehabilitation gloves, enhancing the system’s portability and ease of use. The host part dimensions are 220 mm × 94.15 mm × 34 mm, and the host weighs 476.68 g.

**Figure 3:**
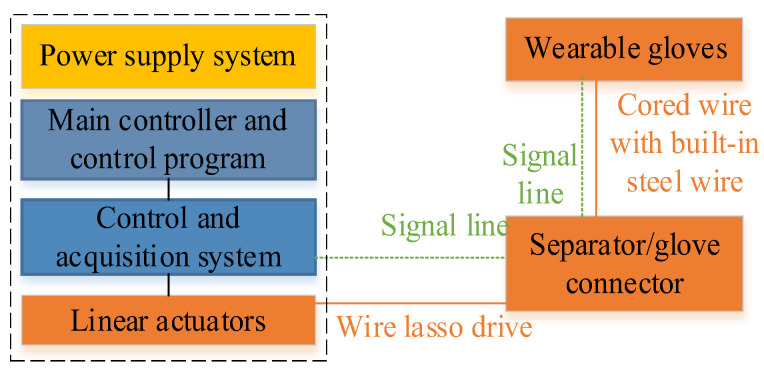
Flexible Glove System Components for sEMG-based Hand Rehabilitation.

The system components are shown in Figure 3. The compact and lightweight design of the wearable glove makes it easy to use for patients, and the system’s portability allows it to be used in various settings, including clinical and home-based settings. The main control module, which includes the linear actuators, battery, hardware, and screen, was specifically designed to be portable and wearable, allowing for a more flexible patient rehabilitation experience. The separation module is an essential system component, effectively combining the central control and the rehabilitation gloves. This approach makes managing and using the system in various settings easier and enhanced its overall portability and ease of use.

The dimensions and weight of the host part were carefully selected to ensure that the wearable glove is compact and lightweight. These features ensure that patients could use the glove comfortably and without undue strain, allowing for the glove to be used over an extended period.

#### 2.2.2. Glove and Finger Structure

**Figure 4:**
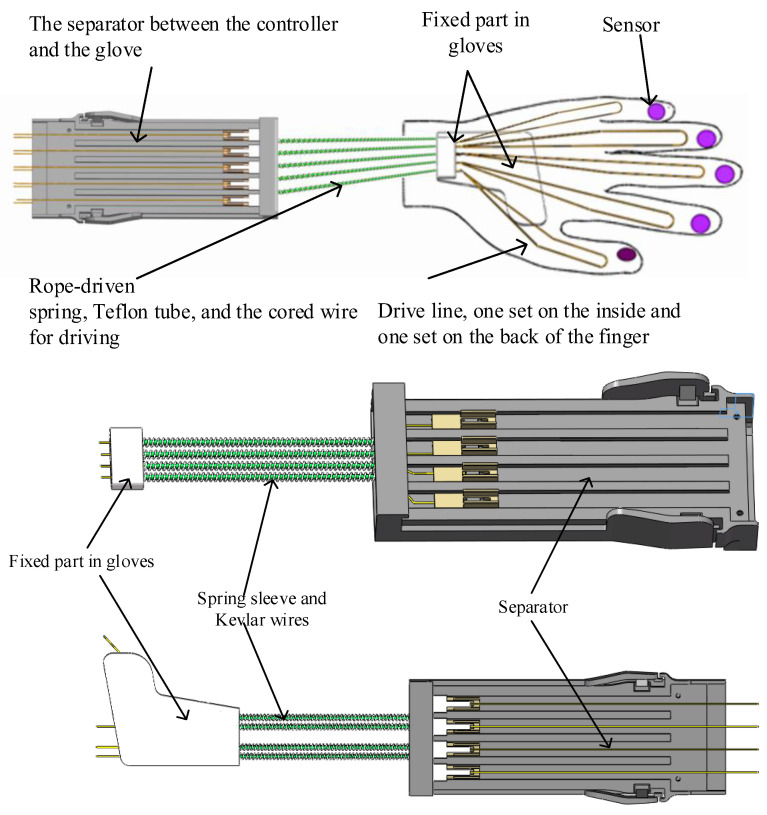
Flexible transmission and glove with sensors.

The structure of the wearable glove used for hand rehabilitation is shown in Figure 4. The outer layer of the glove is composed of a spring tube that remains stationary, while the inner layer is a cored wire with a steel wire lining, which drives the movement of the fingers through the movement of the cored wire. The design incorporates two groups of ropes for each finger, one to drive the finger to bend on the inner side, and the other to drive the finger to straighten on the dorsal side. By pulling the ropes, the glove mimics the natural movement of the fingers, allowing the wearer to perform a range of exercises to improve the motor function. We used a pneumatic tube to reduce friction between the cored wire and the outer spring sleeve. A wire sleeve-fixing structure was used inside the palm. Considering the interference with finger movement, we designed the casing-fixing device and flexible transmission connection as shown in Figure 4.

To monitor the wearer’s progress, pressure sensors were placed at the fingertips to detect the pressure exerted by the fingers during exercises. This information can be used to adjust the glove’s level of assistance, ensuring that the rehabilitation program is tailored to the wearer’s needs.

#### 2.2.3. Driving System with Linear Actuator and Flexible Lasso

**Figure 5:**
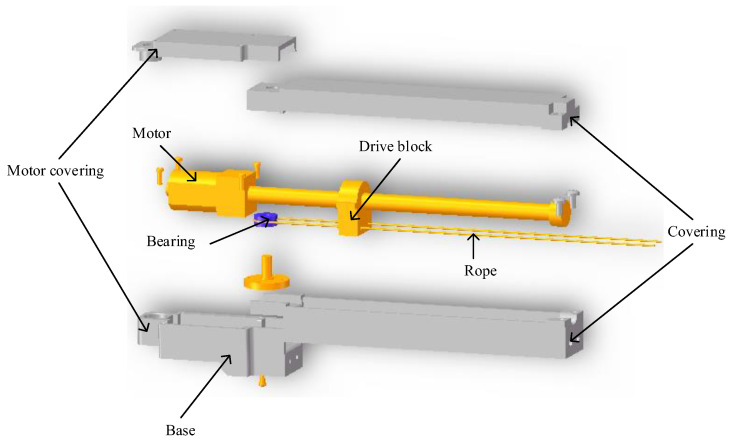
Structure of the linear actuator with bidirectional output.

The wearable glove utilizes linear actuators as a power source to address the need for a portable and compact wearable glove for home-based rehabilitation and daily life assistance. Given that the flexible glove requires bending and straightening each finger, an electric cylinder is not feasible since it can only provide one function. To address this, a specialized linear motor was designed with meshing gears behind the motor reducer, resulting in two outputs in opposite directions, as illustrated in Figure 5. Specifically, the two output sliders move in opposite directions, with one bending the finger by pulling on the inner side of the finger, and the other used to straighten the finger by pulling on the back of the finger. This design ensures the appropriate stroke of each finger for effective rehabilitation and assistive purposes.

**Figure 6:**
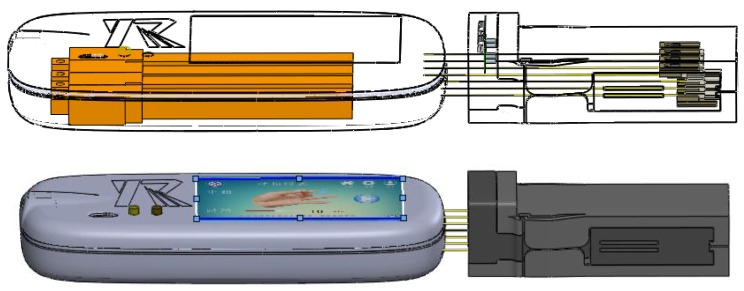
Control machine and separation structure.

The control machine and separation structure are connected flexibly, as depicted in Figure 6. The equipment host contains the motion unit, which is the electric cylinder designed in this study, and each electric cylinder is linked to two flexible transmission lines. The transmission line comprises a spring tube, a plastic tube, and spring steel wire. The outer layer is covered with cloth to transmit power and ensure bending while simplifying the assembly process. The internal execution device consists of a moving wire that pulls a finger back and forth under the action of a linear actuator.

**Figure 7:**
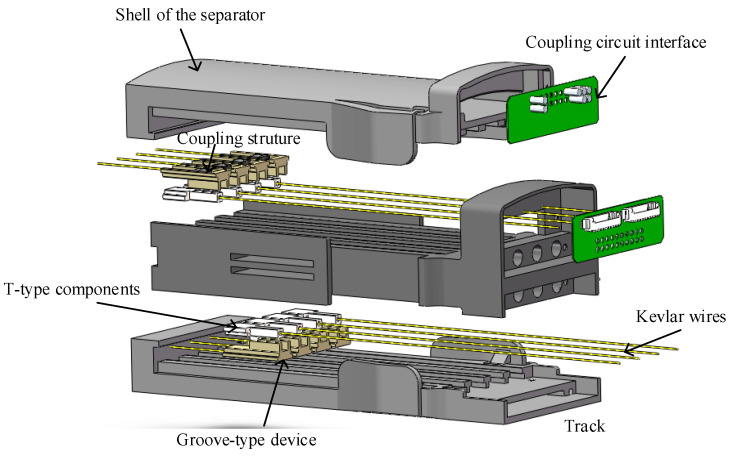
Structure and composition of the separator.

Consider the decoupling design between the central control module and the glove module of wearable rehabilitation robots. In this paper, a separator was designed between the central control module and the glove module to decouple the movement. The splitter makes the signal transmission part of the sensor separable, through spring pins and contacts on the circuit board. The structure and hardware composition of the separator are shown in Figure 7. The motion-coupling structure is completed by a T-type device and a groove-type device. Kevlar wires connect the T-type components and the glove end, and the groove-type components and the central control module are connected by wire. The steel ribbon moves the grooved components, thereby driving the T-shaped components.

#### 2.2.4. Control Protocol of the Hand Robot

**Figure 8:**
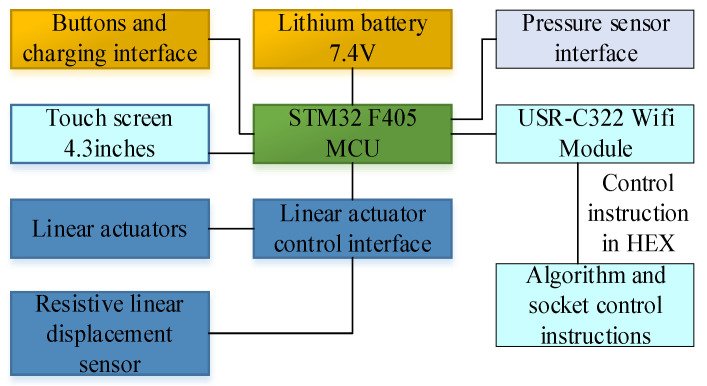
Controller’s hardware components.

The wearable glove controller utilizes the STM32 microcontroller as its central processing unit, with a 12 V lithium battery as the power source for driving the actuators. A serial touch screen enables communication via a serial interface with the central control hardware. Additionally, switch, mode selection, and intensity adjustment buttons were incorporated for selecting the rehabilitation mode during home-based rehabilitation training. A pressure sensor and a resistive linear displacement sensor were integrated into the design to monitor the fingers’ condition and movements within the rehabilitation device. The device is equipped with a lithium battery with a capacity of 11.84 Wh, a standard voltage of 7.4 V, and a nominal capacity of 3200 mAh. During grip mode testing, the power consumption was measured to be 3.7 W, and the device’s continuous exercise time was calculated to be 3.2 h. However, due to the device’s low power consumption during exercise gaps, actual laboratory test rehabilitation training can run for up to 4 h. Figure 8 illustrates the various hardware components of the controller.

**Figure 9:**
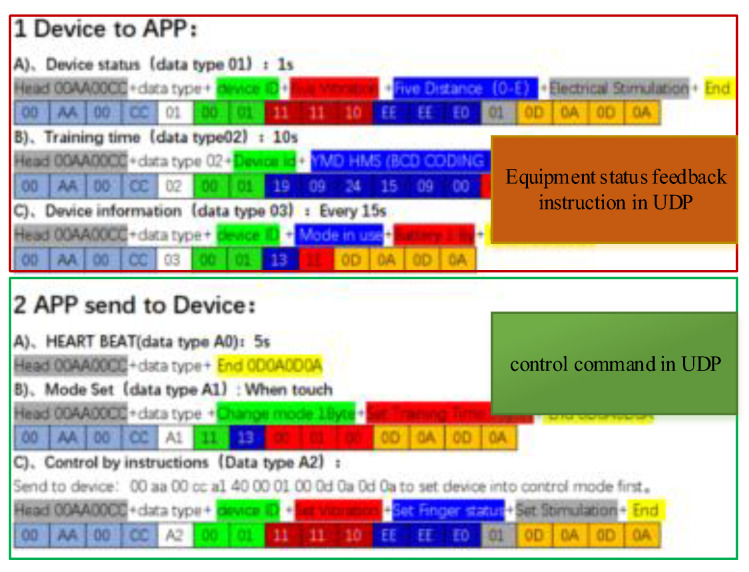
Control instructions of the hand robot.

The WiFi module of USR-C322 was built into the device to communicate with other control modules through UDP. Network communication ensures that the upper computer can control and display the real-time state of the glove, as shown in Figure 9.

Equipment control and equipment status monitoring can be realized through the instructions in the figure above.

The equipment described in this paper mainly uses finger motion control instructions, and the equipment developed and used in this paper does not include vibration and electrical stimulation accessories.

The equipment sends the return instruction of equipment status (data type 0x01) as follows:


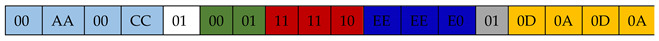


It includes the protocol frame header 0x00AA00CC, data type 0x01, equipment number 0x0001, three bytes used by a five-way vibration motor 0x111110, five-way displacement status 0xEEEEE0, whether electrical stimulation 0x01, frame tail 0x0D0A0D0A.

When we need to control the equipment, we send a control instruction with data type 0xA2 to the corresponding equipment.


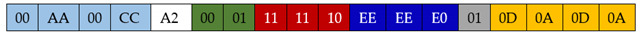


At the same time, we use the following instructions as the heartbeat packet mechanism of the device.

The upper computer sends heartbeat packets (data type A0) to the device regularly:


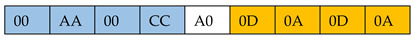


After receiving the heartbeat packet, the device replies to the data with the device number, usage mode, and power information:


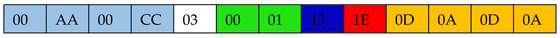


By adding a WiFi module in the development and compiling the program to realize a UDP device control protocol, the device can be controlled by other devices.

### 2.3. Research on Gesture Recognition Algorithms

#### 2.3.1. Gestures and Device Settings

**Figure 10:**
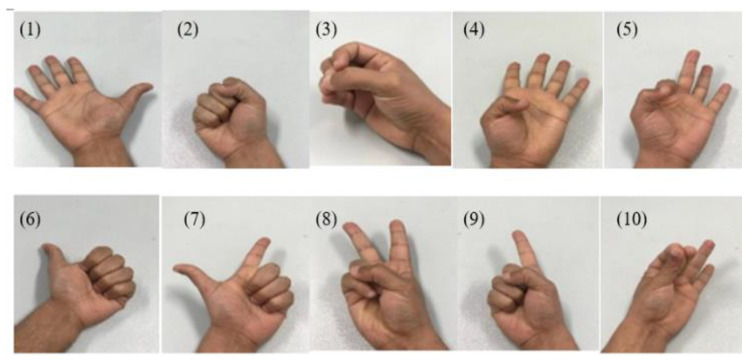
Different gesture designs fit the complexity of the real hand–finger movements.

Gesture recognition was designed to simulate and fit the complexity of real hand-finger movement [31,32,33,34,35,36,37]. Therefore, human hand gesture can be classified into four groups as follows: the first group includes F.H., fisted hand, H.O., hand open, and F.F., finger flexion, and represents different movements of all fingers together. The second group includes the IMRlF, i.e., index, middle, ring, and little fingers, flexion that mimics the good sign, the MRLF—middle, ring, little fingers—flexion, mimicking using a touch screen of an elevator, the TRL—thumb, ring, and little fingers—flexion, mimicking the peace sign, the TMRI—thumb, middle, ring, and little fingers—flexion, mimicking pointing at something, the TIM—thumb, index, and middle fingers—flexion, mimicking grasping some paper. The second group gestures are complex gestures of the hand that exhibit hand flexibility for performing different and complex hand daily movement. The third group includes T.F., thumb, flexed, TIF, thumb and index fingers, flexed to indicate the thumb movement and its cooperation with other fingers. The fourth group includes finger flexion (T.F., IF, M.F., and R.F.), focusing on single finger movements; training can be home-based using task-oriented rehabilitation. These mimicked gestures are shown in Figure 10.

**Figure 11:**
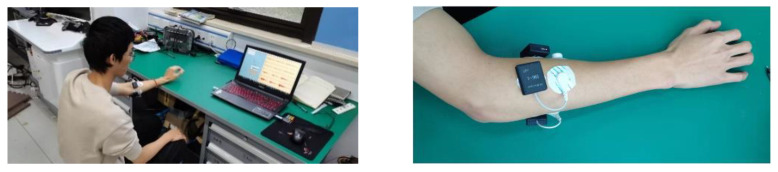
EMG-electrodes placement on the forearm muscles.

A total of eight healthy subjects, six males and two females, all right-handed except one male and between the ages of 21 and 30 years, participated in the data acquisition. The data were collected using (yw-wireless. Beijing Changfeng Technology Co., Ltd., Beijing, China). Four surface electrodes were placed on the forearm muscles, which are four muscles in total (brachioradialis, extensor carpi radialis longus, flexor carpiulnaris, and extensor digitorum), as shown in Figure 11.

**Figure 12:**
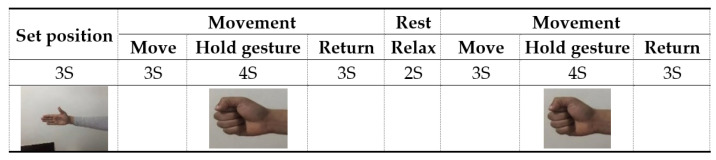
The Setup and the scheduling for the EMG experiment.

sEMG signals were collected at a sampling rate of 1000 Hz, and each gesture was performed 12 times, each time consisting of four runs, each one lasting for 4000 ms; and there was a rest period of 3000 ms to avoid causing muscle fatigue, as shown in Figure 12. The data were collected while maintaining each gesture for 4000 ms, to ensure the presence and the quality of the collected sEMG signals.

This work divided pattern recognition into three main steps: data preprocessing, feature extraction, and pattern recognition. Pattern recognition was divided into two stages, i.e., classifier training and classifier prediction, using new signals from the non-training data.

#### 2.3.2. Data Preprocessing and Feature Extraction

Initially, we selected pertinent data located in the intervals of [(6001:10,000), (18,001:22,000), (30,001:34,000), (42,001:46,000)], which contained EMG signals relevant to the targeted motion. Next, we proceeded with the raw data pre-processing, where the raw signals were trimmed by a butter band-pass filter, ranging from 5 to 480 Hz in areas where an sEMG was present, and further filtered by a notch filter between 48–52 Hz.

To obtain a large set of training data that could be used for pattern recognition training, we used the segmentation overlapped method for extracting useful time-domain features such as mean square value (MAV), root-mean-square (RMS), variance absolute value (VAV), integrated electromyography (Iemg), simple square integral (SSI), waveform length (WL). The extracted featured data contained useful information such as signal strength and amplitude. Later, the wrapper backward method was used to obtain new features. The wrapper method was also used as a feature selection method to select the optimal feature subset by determining the importance of each feature subset independently using a real training test to identify the feature that positively affected the recognition system accuracy. Therefore, we could select the optimal feature subset from the original features. In the feature extraction process, the window length was 200 ms, and the overlap of the adjacent window was 50 ms. The data collected from a single muscle were divided into 317 windows. The 200 ms data window set was a good choice containing enough information to define the contained gesture fully. The 50 ms overlap window utilized the data set to produce a refined, dense classification technique to meet the real-time classification needs.

## 3. Results

### 3.1. Accuracy of Gesture Recognition

**Figure 13:**
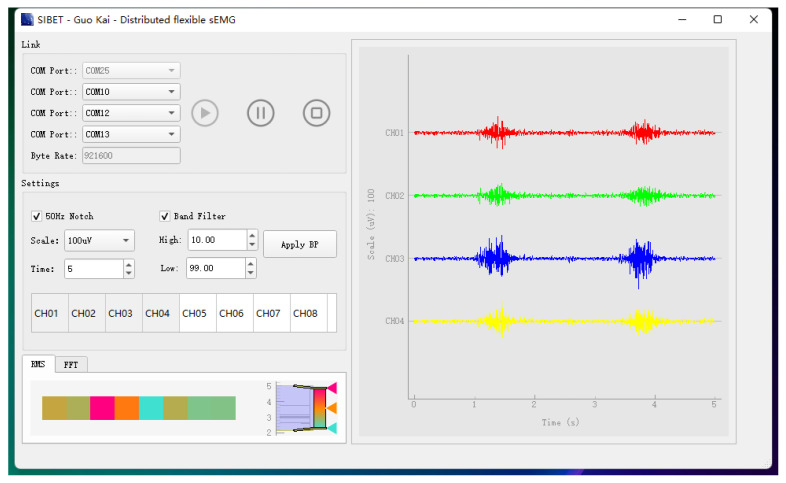
Software interface of four lead EMG.

This article used the PyQt5 framework to build software for EMG acquisition and raw data storage, as shown in Figure 13. In real time, the software effectively displayed the four lead EMG signals alongside the real-time predicted gestures, providing accurate results to the users.

**Figure 14:**
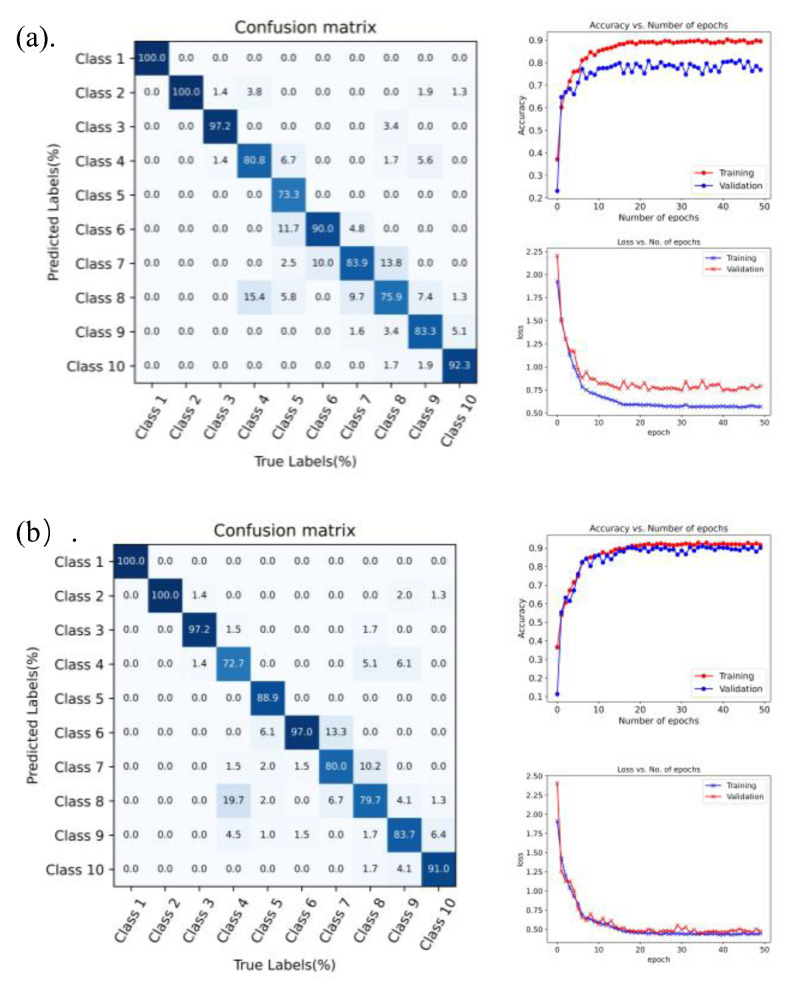
Confusion matrix and iteration accuracy of different algorithms, (**a**) using the 1D-CNN algorithm, (**b**) using the InceptionTime algorithm.

We adopted the 1D-CNN algorithm and the InceptionTime algorithm. The above-collected data were intelligently identified. The obtained iteration accuracy and confusion matrix of ten gestures are shown in Figure 14.

Figure 14 shows the accuracy achieved by using the two algorithms and the accuracy for specific gestures. In Figure 14a, we adopted the 1D-CNN algorithm, achieving an accuracy of 89.52% in the training set and of 76.84% in the verification set, with an overall accuracy of 80.98%. In Figure 14b, we adopted the InceptionTime algorithm, achieving an accuracy of 91.60% in the training set and of 90.09% in the verification set, with an overall accuracy of 90.89%.

### 3.2. Control of the Hand Rehabilitation Robot Based on sEMG

**Figure 15:**
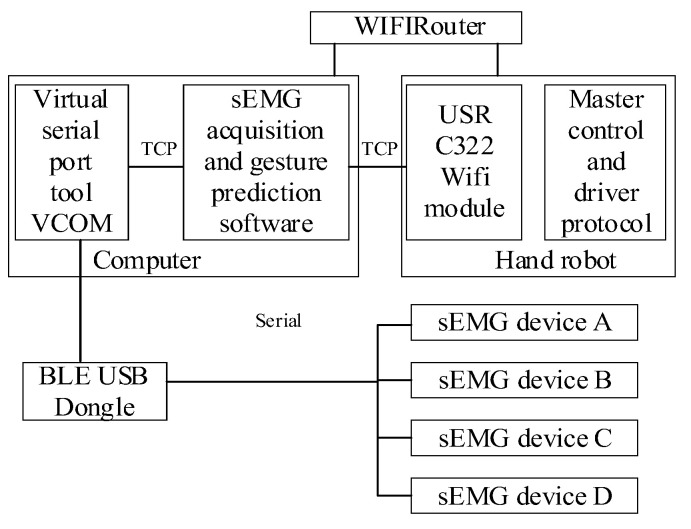
Framework of communication and control.

The sEMG drive of the hand rehabilitation robot using the framework is shown in Figure 15.

**Figure 16:**
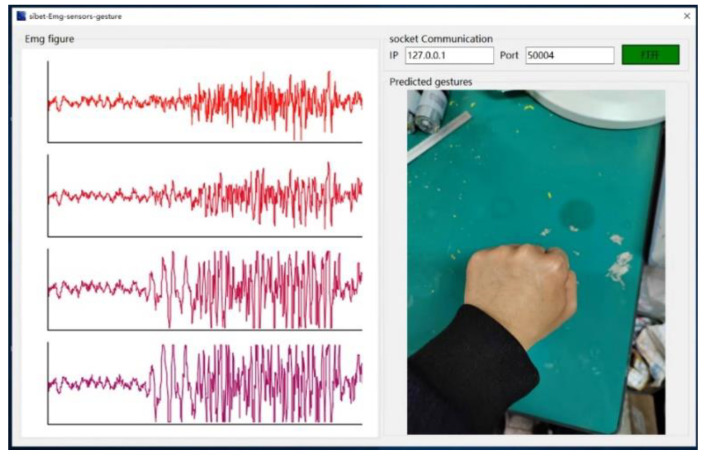
Online recognition software for grasping and stretching.

Based on the above framework, we developed an online recognition software for grasping and extending a hand using the above algorithm, as shown in Figure 16. We implemented the control of the rehabilitation robot based on this interface.

**Figure 17:**
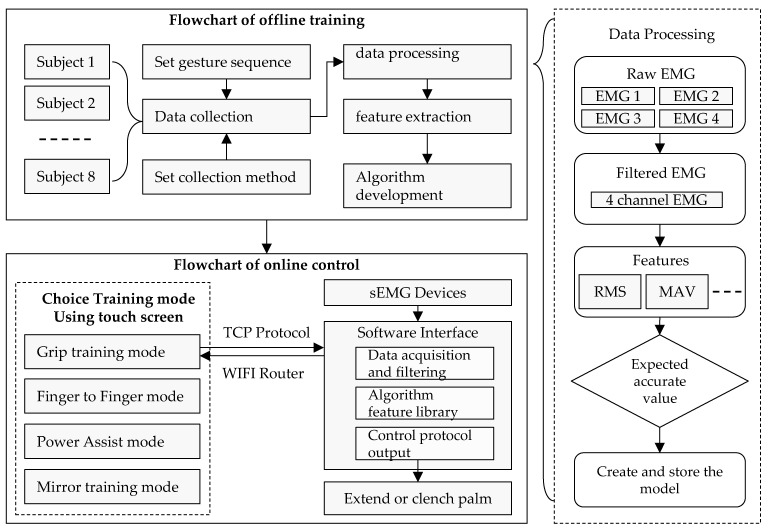
Flowchart of the hand robot control with sEMG.

The corresponding flowchart is shown in Figure 17. The algorithm was developed with the help of the eight people, and data collection was completed after setting gesture sequences and collection methods. Subsequently, data filtering and other preprocessing operations were carried out, and relevant features in the time and frequency domains were extracted, ultimately completing the development of the algorithm. For the online control of robots, the first step is to select the mirror mode through a screen, and the computer and device communicate through the TCP protocol. After obtaining electromyographic data through the computer software, the data were processed and algorithmically recognized, ultimately outputting the motion instructions for the robot.

**Figure 18:**
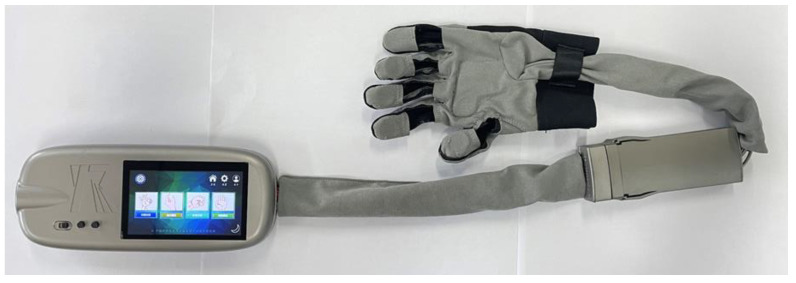
Hand rehabilitation robot that was developed in this study.

The complete hand rehabilitation robot we constructed as described in this study is shown in Figure 18.

**Figure 19:**
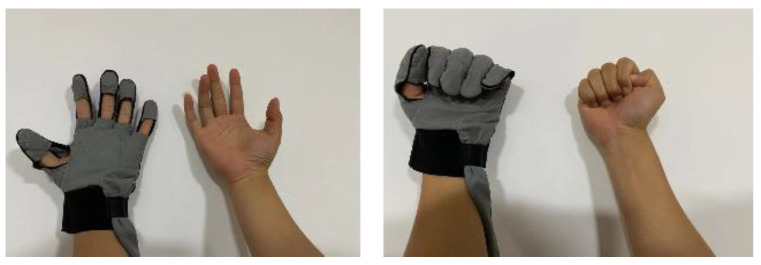
Use of the sEMG device and software for mirror therapy.

The control of the rehabilitation robot was realized by sEMG driving, as shown in Figure 19. This Figure also shows different snapshots of the affected hand assisted by the wearable glove.

## 4. Discussion

A highly accurate decoding and classification method for sEMG signals was developed in this research, resulting in a classification accuracy of 90.89%. The contribution of this work surpasses that of previous hand rehabilitation systems. The wearable glove that was created can accurately perform ten desired motions, including six task-oriented therapy-based motions. Additionally, the glove is inexpensive and exceptionally comfortable. It can be used as a home-based rehabilitation system and is not limited to clinical environments. The glove can train individual finger movements and multiple finger cooperation, with a latency time of less than 300 ms, thus satisfying online training requirements. The controller, glove, and glove structure were designed to align with human–machine integration and ergonomics, as hand-function robots are used for rehabilitation and muscle strength enhancement. The host structure has no edges or sharp corners, with round edges made of metallic silver. The wearable device mainly operates through a touch screen, offering good man–machine interaction characteristics.

The core actuator of the robot was designed independently and can output a two-way rope-pulling force. Generally, the rope can only transmit the pulling force, and two actuators are needed to realize a bidirectional drive. In this paper, the actuator has two reverse outputs; so, a single actuator can directly drive a finger forward and backward. The transmission adopts an optimized lasso transmission design, which is closer to the movement characteristics of human muscles. Compared with other flexible drives, the lasso drive has the characteristics of fine-grained operation, reliable transmission, and high safety. In this paper, the lasso drive presents a single-tube bidirectional lasso that can realize a finger’s flexion and extension.

The glove part considers the palm movement, a challenge for most wearable hand devices, and the design is lighter and more convenient to wear. A flexible thin-film pressure sensor was placed at the fingertips of the glove to collect pressure intended for muscle strength enhancement. The driving rope was sewn into the glove, obtaining a solution close to the real driving force of the muscle. The whole machine has a WIFI communication interface that grants intelligent control through the protocol.

In the offline training, the InceptionTime algorithm had the highest accuracy among all the algorithms. It showed less prediction time and required less training time than the 1D-CNN algorithm, and the average difference in accuracy between them was ±9.2%. We decided to use the InceptionTime algorithm for its low prediction time, which is important for our application. The highest recorded accuracy was 90.89%, which satisfies our wearable glove application.

## 5. Conclusions

In conclusion, the presented wearable hand rehabilitation system has the potential to significantly impact the motor recovery of paresis and hemiparesis patients. Actuated gloves with sEMG sensing are a flexible, wearable technology that is safe, comfortable, and portable. Additionally, the system can be affordable for low- and middle-income countries, which have limited access to rigid exoskeleton devices due to their high cost. Compared to dedicated-data gloves with targeted sensors, the wearable glove utilizing biomedical signals offers improved signal quality and higher accuracy in detecting the desired motions during rehabilitation training. This connection between the desired motion activated by the brain and sent to the muscle and the actual movement executed by the actuated glove shown on a screen can speed up the rehabilitation process based on the mirror therapy technique. However, it is worth noting that achieving a fine-grained classification of the training gestures still requires the precise placement of the EMG electrode. This study presents a promising wearable rehabilitation system for hand motor recovery that can be used in various settings, including clinical and home-based environments.

## Data Availability

The data presented in this study are available on request from the corresponding author.
